# Burnout among Iranian nurses: a national survey

**DOI:** 10.1186/s12912-020-00461-7

**Published:** 2020-07-16

**Authors:** Sara Mahmoudi, Maasoumeh Barkhordari-Sharifabad, Amir-Hosein Pishgooie, Foroozan Atashzadeh-Shoorideh, Zahra Lotfi

**Affiliations:** 1 Department of Nursing, School of Nursing, Dezful University of Medical Sciences, Dezful, Iran; 2 Department of Nursing, School of Medical Science, Yazd Branch, Islamic Azad University, Yazd, Iran; 3 Department of Critical Care Nursing, School of Nursing, AJA University of Medical Sciences, Tehran, Iran; 4 Department of Psychiatric Nursing and Management, School of Nursing & Midwifery, Shahid Beheshti University of Medical Sciences, Vali-Asr Avenue, Cross of Vali-Asr and Hashemi Rafsanjani Highway, Opposite to Rajaee Heart Hospital, Tehran, Iran; 5 Department of Nursing, Royal Free Hospital, London, UK

**Keywords:** Critical care, Medical, Surgical, Burnout, Nurse

## Abstract

**Background:**

Nurses, particularly critical care nurses, are exposed to high levels of stress and burnout. Burnout is associated with many deleterious consequences affecting health care outcomes. The present study is intended to determine the dimensions of burnout in nurses on surgical, medical and critical care units and its relationship with demographic characteristics.

**Methods:**

In this descriptive research study, performed at critical and non- critical care units, 743 nurses were randomly selected by quota sampling from medical sciences universities in Iran. Data collection instruments included a “demographic questionnaire” and the “Persian version of the Copenhagen Burnout Inventory. Data were analyzed using SPSS20.

**Results:**

The findings showed that regarding all dimensions, the lowest level of burnout belonged to surgical wards whereas the highest level pertained to critical care wards indicating a significant difference among various aspects of burnout in different wards, i.e., surgery, medical, and critical care. There was no significant difference in gender, academic degree, and marital status in any of the aspects of burnout in critical care units; yet, the difference was significant between surgical and medical wards (*P* < 0.05). There were a negative significant correlation between some dimensions of burnout with age and nursing experience in critical care and medical wards (*P* < 0.05). Whereas in surgical wards, there were a positive significant correlation between some aspects of burnout with nursing experience and age (*P* < 0.05).

**Conclusion:**

This study found that the critical care nurses have significantly higher level of burnout compared to the medical-surgical nurses. These results should be considered when planning burnout prevention schedules for nurses.

## Background

Burnout is defined by Kristensen as the physical and mental exhaustion experienced by everyone [1]. Burnout may be identified by loss of energy, which commonly occurs when people feel physically and mentally “exhausted” [2], and has three aspects: personal, work-related, and client-related [1]. On the other hand, burnout is a long and continued reaction to prolonged emotional and interpersonal job stressors and stressful events that can influence both personal and organizational aspects [3–6]. As shown in many studies, burnout is a progressively significant public health problem, which has an important effect on the nurses’ health and well-being, and the quantity and quality of patients care [7]. Additionally, it is also correlated to a mass of psychosomatic problems and weakened quality of life [8], and may influence psychological and physical disorders [9, 10]. Moreover, burnout can have negative effects on the nurses’ practice, patient safety [11, 12], and patients’ satisfaction; also, it can increase the likelihood of work-related injuries and subsequent absence from work [12]. It also affects job ssatisfaction, and nurse turnover [13]. In fact, nurses’ burnout not only threatens their own condition and health, but also impacts on the quality of care given to patients [8]. Burnout appears to differ across the different nursing specialties [14] and differences in burnout across work settings is not unique [15]. Regarding nursing specialties, acute and/or critical care nurses look to be at a significantly higher risk of burnout [14] as a result of the extremely demanding situation in emergency medicine (EM), hemodialysis (HD) and in intensive care units (ICU) [7].

Nursing shortage, lack of support at work, caring for very unwell patients, and perceived conflicts with patients or other members of staff and high workload are some of the reasons for occurrence of burnout in nurses. Also, the high level of burnout is connected to lack of satisfaction with wages, opportunity for advancement, study leave and a practice setting with insufficient staffing as well as resources, and absence of nurses’ contribution in hospital management [16, 17].

Conditions such as these in Iran have led to nurses facing an increased probability of physical and psychological stress, which may cause burnout [18]. The results of several studies on burnout revealed that burnout prevalence among Iranian nurses is high [19]. Different studies have been carried out on stress and burnout among nurses in the general profession, but few studies were conducted among acute and critical care nurses [20]. There are a number of studies on burnout in Iran most of which have focused on general specialist nurses, occupational therapists, and psychologists. The findings of the studies on burnout in hospital nurses were controversial. A comparison of medical, surgery, psychiatry, and burns wards showed that nurses of psychiatry wards had meaningfully higher levels of emotional exhaustion and depersonalization compared to nurses working in other wards; burns wards nurses had significantly higher levels of personal accomplishment [21]. The authors did not find any research about burnout in emergency and hemodialysis specialist nurses in the Iranian context.

Besides, what looks more important is to measure the concept effectively, and to do so, a reliable validated scale is required. The Copenhagen Burnout Inventory (CBI) sufficiently measures the potential analyses of etiology and the results of burnout and workplace interventions [22]. The CBI has been well practical in health and human service centers [23].

### Aim

This study will investigate the dimensions of burnout in nurses working in surgical, medical and critical care units, and its relationship with demographic characteristics.

## Method

### Research design, participants and setting

In this descriptive-analytical study, the participants were selected by quota sampling. A multistage randomized sampling used for increasing generalizability. Medical science university hospitals in Iran were divided to five areas as north, south, east, west and center. The mean number of beds in each hospital is 210. After random selection of hospitals in every area (15 hospitals), based on Cochrane sample size determination formula with 95% confidence level, 2% Confidence Interval and population of 1158 nurses, the sample size was calculated about 781. Thirty-eight questionnaire were not returned and finally 743 questionnaires were collected. The inclusion criterion was: having at least 6 m of work experience as a member of nursing staff in the current clinical wards.

### Variable and instruments

In this study, a demographic and occupational questionnaire and Persian Copenhagen Burnout Inventory (P-CBI) were used to collect the data.

#### 1- Demographic and occupational questionnaire

For the demographic data, the participants were asked to state their age, gender, educational level, years of nursing experience, marital status.

#### 2- Persian Copenhagen burnout inventory (P-CBI)

The four-factor P-CBI contains 19 items and uses a 5-item Likert-type scale. Items have responses of frequency ranging from; 100 (always or very high degree), 75 (often or high degree), 50 (sometimes or somewhat), 25 (seldom or low degree) and 0 (never or very low degree). It includes four subscales of personal burnout (7 items), nature of work-related burnout (3 items), work aversion-related burnout (3 items) and client-related burnout (6 items). Use of these four subscales is specific for the Persian version of CBI. Total score on the scale is the average of the scores on the items. Cronbach’s α coefficient for its subscales after factor analysis ranged between 0.84 and 0.89. The test-retest reliability coefficients by the Intra-Class Coefficient correlation for subscales of personal burnout, nature of work-related burnout, work aversion-related burnout, as well as client-related burnout were reported as 0.95, 0.84, 0.83, and 0.90 (*P* < 0.001), respectively. Higher scores for each sub-scale signified more burnout. No cutting-point or reference values exist for this scale [24].

### Data collection

Collecting data was completed from May to September 2017. The questionnaires were distributed to the nurses after the first author had explained the goals of the research. Nurses were asked to respond at a convenient time, when they were not busy. Each questionnaire contained explanatory details about the survey, and a reply envelope was supplied. The nurses were asked to place their responses into the reply envelopes. The questionnaires were collected after 2 days.

### Ethical considerations

Ethical approval and agreement were obtained from the Research and Ethics Committees of University of Medical Sciences. Informed written consents was also acquired from all the participants who took part in the study. All ethical issues of respondents’ anonymity and confidentiality were observed in this study.

### Statistical analysis

Statistical analyses were performed with SPSS20. Mean, standard deviation (SD) and frequency were presented to describe the variables. Independent-sample t-test and ANOVA were used when comparing groups on normally distributed variables.

## Results

Of 743 participants of the study, 282 (37.95%) were employed in the surgical ward, 302 (40.64%) in the medical ward, and 159 (21.39%) in the critical care wards. The total number of critical care wards was smaller than other wards. The greatest mean age was 33.81 ± 8.09 years and the greatest mean work experience was 9.78 ± 7.22 years that pertained to the surgical ward. There was no significant difference in age, work experience, marital status and academic degree among the wards. In critical care units there were more males than in medical and surgical ones (Table 1).

**Table 1:** Demographic and occupational characteristics of the participants

Variable		Ward	***N*** (%)	Mean (SD)	***P***
**Age**		Surgical		33.81 (8.09)	.11
		Medical		32.68 (6.62)	
		Critical care		33.75 (6.50)	
		Total		33.34 (7.20)	
**Nursing experience**		Surgical		9.78 (7.22)	.55
		Medical		9.19 (6.29)	
		Critical care		9.53 (5.95)	
		Total		9.49 (6.58)	
**Gender**	**Male**	Surgical	27 (9.57)		< 0.001
		Medical	30 (9.93)		
		Critical care	44 (27.67)		
		Total	101 (13.59)		
	**Female**	Surgical	255 (90.43)		
		Medical	272 (90.07)		
		Critical care	115 (72.33)		
		Total	642 (86.41)		
**Nurse’s educational level**	**Bachelor of Science**	Surgical	263 (93.26)		.59
		Medical	284 (94.04)		
		Critical care	152 (95.60)		
		Total	699 (94.08)		
	**Master of Science**	Surgical	19 (6.74)		
		Medical	18 (5.96)		
		Critical care	7 (4.40)		
		Total	44 (5.92)		
**Marital status**	**Single**	Surgical	108 (38.30)		.34
		Medical	109 (36.0)		
		Critical care	57 (35.85)		
		Total	274 (36.88)		
	**married**	Surgical	165 (58.50)		
		Medical	190 (62.91)		
		Critical care	99 (62.26)		
		Total	454 (61.10)		
	**Divorce**	Surgical	6 (2.10)		
		Medical	3 (0.99)		
		Critical care	2 (1.26)		
		Total	11 (1.49)		
	**Widow**	Surgical	3 (1.10)		
		Medical	0 (0.00)		
		Critical care	0 (0.00)		
		Total	3 (0.40)		
	**Living alone**	Surgical	0 (0.00)		
		Medical	0 (0.00)		
		Critical care	1 (0.63)		
		Total	1 (0.13)		

Also, the findings indicated that among all dimensions of burnout, the lowest mean belonged to the surgical ward whereas the highest mean pertained to the critical care ward. There was a significant difference in various dimensions of burnout among different wards (Table 2).

**Table 2:** Dimensions of occupational burnout among different wards

Dimensions of burnout	Unit	Mean (SD)	Max	Min	F	***P***
**Personal burnout**	Surgical	46.31 (19.39)	78.58	14.29	86.99	< 0.001
	Medical	60.40 (18.69)	100	28.57		
	Critical care	69.85 (18.27)	100	25		
	Total	57.08 (20.94)	100	14.29		
**Nature work-related burnout**	Surgical	51.32 (22.04)	83.33	0	71.04	< 0.001
	Medical	57.91 (19.79)	100	8.33		
	Critical care	76.04 (21.66)	100	25		
	Total	59.29 (22.97)	100	0		
**Work aversion-related burnout**	Surgical	37.58 (13.73)	66.67	8.33	236.43	< 0.001
	Medical	43.47 (23.86)	100	0		
	Critical care	76.10 (13.65)	100	33.33		
	Total	48.22 (23.68)	100	0		
**Client-related burnout**	Surgical	45.36 (22.65)	100	8.33	114.60	< 0.001
	Medical	63.71 (21.16)	100	63.71		
	Critical care	75.44 (17.90)	100	25		
	Total	59.25 (24.12)	100	8.33		

The results further suggested that regarding personal burnout in males, the highest mean belonged to the medical ward whereas it pertained to critical care wards in the three other dimensions. In females, the highest mean of personal burnout pertained to critical care wards in all aspects. T-test demonstrated no significant difference between males and females in ICU wards in all aspects of burnout though, albeit, the difference was significant in other wards (Table 3). The results of T-test also suggested no significant difference in all dimensions of burnout in critical care ward with respect to academic degree, though the difference was significant in other wards so that nurses with a BS degree showed greater burnout (Table 3).

**Table 3:** Relationship of Dimensions of burnout in different wards in terms of demographic and occupational characteristics

Dimensions of burnout	Personal Burnout			Nature work-related burnout			Work aversion-related burnout			Client-related burnout		
Demographic and occupational characteristics	Surgical	Medical	Critical Care	Surgical	Medical	Critical Care	Surgical	Medical	Critical Care	Surgical	Medical	Critical Care
**Gender Mean (SD)**												
**Male**	36.50 (18.13)	74.28 (15.47)	69.15 (18.37)	43.51 (16.87)	70.00 (17.45)	75.37 (21.78)	32.40 (9.55)	53.75 (27.62)	76.32 (13.32)	37.03 (13.34)	74.16 (14.89)	76.23 (15.81)
**Female**	47.35 (19.26)	58.87 (18.40)	70.12 (18.31)	52.15 (22.38)	56.58 (19.61)	76.30 (21.70)	38.13 (14.00)	42.34 (23.16)	76.01 (13.83)	46.24 (23.27)	62.56 (21.45)	75.14 (18.69)
***P*-value**	< 0.01	< 0.001	.76	< 0.05	< 0.001	.81	< 0.001	< 0.05	.89	< 0.01	< 0.01	.73
**Nurse’s educational level Mean (SD)**												
**Bachelor of Science**	47.73 (19.21)	61.14 (18.54)	70.39 (17.49)	52.97 (21.62)	59.03 (18.88)	76.69 (20.93)	38.92 (13.05)	44.42 (23.57)	75.98 (13.28)	47.37 (21.90)	65.19 (19.71)	75.90 (17.58)
**Master of Science**	26.69 (7.92)	48.80 (17.58)	58.16 (30.49)	28.50 (13.96)	40.27 (25.44)	61.90 (32.93)	19.07 (8.70)	28.47 (24.05)	78.57 (21.43)	17.54 (12.38)	40.27 (29.04)	65.47 (23.16)
***P*-value**	< 0.001	< 0.01	.33	< 0.001	< 0.01	.28	< 0.001	< 0.01	0.62	< 0.00	< 0.001	.13
**Marital status Mean (SD)**												
**single**	38.26 (19.03)	58.71 (17.42)	70.42 (17.19)	43.28 (18.28)	54.96 (21.12)	78.07 (21.22)	32.83 (13.17)	40.86 (24.31)	74.85 (13.12)	36.65 (16.38)	58.18 (22.62)	74.56 (16.84)
**married**	52.22 (17.94)	61.25 (19.48)	69.80 (18.44)	57.27 (22.95)	59.34 (18.90)	75.00 (22.11)	41.99 (41.99)	44.67 (23.62)	76.43 (13.93)	52.17 (24.03)	66.57 (19.71)	75.71 (18.50)
**divorced**	35.71 (0.00)	67.85 (0.00)	76.78 (32.82)	41.66 (0.00)	75.00 (0.00)	79.16 (17.67)	16.66 (0.00)	62.50 (0.00)	91.66 (11.78)	33.33 (0.00)	83.33 (0.00)	95.83 (5.89)
**widow**	32.14 (0.00)			33.33 (0.00)			8.33 (0.00)			8.33 (0.00)		
**Living alone**			28.57 (0.00)			58.33 (0.00)			83.33 (0.00)			58.33 (0.00)
***P*-value**	< 0.001	0.41	0.14	< 0.001	0.05	0.70	< 0.001	0.15	0.33	< 0.001	< 0.001	0.30
**Age**												
**r**	0.11	−0.009	−0.4	0.12	−0.35	−0.39	0.01	−0.13	−0.11	0.004	−0.05	−0.35
***P*-value**	0.06	0.88	< 0.001	0.03	0.54	< 0.001	0.75	0.02	0.15	0.94	0.35	< 0.001
**Nursing experience**												
**r**	0.13	0.005	−0.35	0.12	0.002	−0.34	−0.008	− 0.12	−0.14	− 0.02	−0.03	− 0.34
***P*-value**	0.01	0.92	< 0.001	0.04	0.96	< 0.001	0.88	0.03	0.06	0.63	0.49	< 0.001

Additionally, the findings indicated that the highest mean pertained to critical care wards with respect to marital status in all aspects of burnout. Of course, the results of ANOVA indicated that marital status exerted no significant effect on burnout in critical care wards, though the difference was significant in all aspects in surgical ward and married group had highest mean of burnout. Also, the difference was significant in client-related burnout in medical ward with respect to marital status as being divorced exerted a significant effect on burnout level (Table 3).

There were a negative correlation between dimensions of “personal burnout” and “Work aversion-related burnout” and “client-related burnout” with age and nursing experience in critical care wards. In surgical wards, there were a positive correlation between dimension of “personal burnout” and nursing experience and also a positive correlation between dimension of “nature work-related burnout” with nursing experience and age. Results of correlation test showed that there are a negative correlation between dimension of “work aversion-related burnout” and age and nursing experience in medical wards (Table 3).

## Discussion

This study determined the dimensions of burnout in nursing staffs of surgical, medical and critical care units and its relationship with demographic characteristics.

The results indicated that among all aspects of burnout, the lowest mean belonged to surgical ward whereas the highest mean pertained to critical care ward. There was a significant difference in all aspects of burnout among various wards. Numerous similar studies have been conducted on nurses working in different wards. Nonetheless, the use of different instruments and unusual study samples makes comparison of results difficult.

Scholars have presented different findings on the extent of burnout in different units in Iran. Similar to the findings of the present study, Salimi et al. found high level of burnout and compassion fatigue among 400 nurses working in the intensive care units of Iranian hospitals [25]. In the study of Saharian et al., on 180 nurses from 5 hospitals in Shiraz/Iran, Nurses in psychiatric wards had higher levels of emotional burnout and depersonalization, and burn ward nurses had higher rates of personal accomplishment [26]. Another study on 100 nurses working in different units in Rudsar/Iran indicated that ER nurses had higher job burnout than other nurses [27].

Similar results have been reported in other countries. Study of systematic review on ER nurses indicated a high level of occupational burnout among them so that, on the average, 26% of ER nurses suffer from burnout. Scholars have reported the mean weight percentage of respondents above the cut-off point as 26% for emotional burnout, 35% for depersonalization, and 27% for lack of personal accomplishment [28]. In addition, the results of the study, in the Małopolska region, indicated individuals employed in ICU wards have higher emotional burnout, higher depersonalization, and lower personal accomplishment. However, multidimensional model of analysis showed a negative significant correlation between working in ICU ward and depersonalization in their study. Indeed, working in ICU ward served as a protective factor against burnout for nurses due to imagination of awards (being more professional) [29]. Ghazanfar et al. reported similar findings in cardiac care units in Pakistan [30]. Other studies also showed that HD nurses were exposed to high levels of stress and moderate burnout [31, 32]. Another study findings suggested moderate-to-high levels of occupational burnout in nurses at various wards of hospital; yet, contrary to our findings, there was no statistically significant difference among nurses working in ICU, ER, and surgical wards [33].

The disparity in results may be attributed to the different instruments used by the studies, study samples or population, and the organizational structure used. It can be related to the prevailing conditions in the organization and the environmental conditions. In other words, patients referred to ICU are usually physically in critical condition. In fact Iranian ICU nurses in addition to tolerating the pervasive psychosocial pressures prevailing in all parts of the hospital such as nursing shortages, job dissatisfaction, poor social position of nurses and etc., they face certain pressures, such as the urgency of time, exposure to the critical condition of patients s with poor prognosis, which in turn leads to negative emotions.

Furthermore, according to the study which was conducted in this regard, technology use in the organizational structure may be a stressful element. Consequently, by the growing use of technology, stress level rises in the workplace and professions that deal with modern technologies in critical care unit where nurses are exposed to higher workload and stress which can result in high level of burnout [34]. As a final point, critical nurses have to conduct various demanding roles such as advocator, caregiver, teacher, counselor and technician while patients stay in a critical care unit. These complex roles undertaken by these nurses in addition to organizational aspects of the work situation have led to critical nurses experiencing high levels of burnout.

It should be noted that in many cities of Iran, hospitals are small and there is not enough beds. For this reason, patients with neurological, nephrological, cardiac, and other problems are admitted to the medical ward. Patients admitted to the surgical ward are also candidates for various surgical procedures.

The results indicated that in both genders, critical care nurses suffered from greater burnout with no significant difference among them. In surgical wards, women showed the greatest level of burnout in all aspects whereas in medical wards, men showed the highest levels of burnout indicating a significant difference between the two genders. The results of numerous studies demonstrated higher occupational burnout among males than females [31, 35]. Because of the patient’s status in men’s medical ward, nurses are exposed to more stress. There is a lot of evidence that patients hospitalized in the medical wards suffer from increased anxiety and depression due to the chronic nature of their illness. This can increase the vulnerability of nurses working in these wards [27, 36, 37]. On the other hand, work in the medical units is routine, uniform and unprofessional (the lack of specialized job duties), and this is unattractive for men and therefore causes more burnout. However, displacement and the multiplicity of work such as dressing, frequent check-ups of vital signs and severe shortage of manpower in surgical wards have led to greater burnout of women in surgical wards.

The results of the study further revealed that the highest level of burnout in all aspects in nurses holding a BS or MSc degree belonged to critical care wards; nevertheless, there was no significant difference in all aspects with respect to different academic degrees in critical care wards, yet, in surgical and medical wards, all aspects were significantly different in nurses holding a BS degree. The findings of the study by Demir suggested that higher academic degrees and higher occupational positions reduce occupational burnout [38]. The study by Rashedi et al. showed inadequate professional training leads to burnout in Iranian nurses [39]. Higher burnout in nurses with a BS degree can be due to insufficient education on how to manage work problems and increase resilience in the Iranian nursing education system. On the other hand, in the medical and surgical units, nurses by earning a higher degree, can benefit from higher job opportunities such as higher salaries, reduced work hours, and higher job qualifications in the future. For ICU nurses, because of the specialization of nursing care, it is possible to showcase their abilities and gain positive attitudes about themselves and patients; and while feeling confident, they will have a greater sense of power and mastery over their tasks.

Furthermore, the results indicated that marital status had no effect on burnout in critical care wards and the aspects were the same for all marital groups. Various studies have found no relationship between marital status and burnout in nurses and this lack of relationship was attributed to the lower probability of personal life in the occurrence of occupational burnout [37, 40, 41]. Unlike the results of the present study, one study demonstrated that particular variables, for example being single, are recognized as elements that may be related to high level of burnout in critical care nurses [34].

It should be pointed out that, in the present study, there was a significant difference in all aspects of burnout according to marital status in surgical wards and married group had highest mean of burnout. Family responsibilities, along with the variety of work and patients in the surgical ward, can be the cause of more burnout in all dimensions in surgical nurses.

Also, the difference was significant in client-related burnout in medical ward and being divorced exerted a significant effect on burnout level. Family-work conflict can be effective in developing burnout [42]. Studies have shown that family-work conflict and family composition are important determinants of burnout [43]. Anxiety and depression due to the chronic disease of patients hospitalized in the medical wards is associated with higher scores for client-related burnout [27, 36, 37]. Also, stressors arising from conflict with patients can lead to higher the client-related burnout [44].

Results showed there were a negative correlation between dimensions of “personal burnout” and “work aversion-related burnout” and “client-related burnout” with age and nursing experience in critical care wards. Studies are showed an increase in age is related to a decrease in reported burnout [45] People with low age and work experience become more stressed and feeling less self-sufficient due to lack of sufficient work experience and fear of error in critical situations [37].

In surgical wards, there were a positive correlation between dimension of “personal burnout” and nursing experience and also a positive correlation between dimension of “nature work-related burnout” with nursing experience and age. Study of Low et al. showed that older age of surgery residents was significantly associated with higher burnout [46].

Also, results showed that there are a negative correlation between dimension of “work aversion-related burnout” and age and nursing experience in medical wards. These association can be explained by the using of effective coping strategies by experienced nurses [39, 47, 48].

### Clinical implications of the results

This study helps identification of high risk groups and risk factors for developing effective preventive strategies by identifying the dimensions of burnout and occupational and demographic characteristics in different units. These results should be considered when planning burnout prevention schedules for critical care nurses. Conducting some supportive interventions such as improved support from nurse managers and colleagues can act as a buffer against the stressors of workloads, the intense personal relationship, and repeated exposures to patient death. As responsible persons in charge of the emotional cost of the job, nurse managers have to actively ease psychological and spiritual support of nurses.

Formal programs such as mentoring, formal collegial support and increased nurse manager support promote psychological well-being and team cohesiveness [49]. Regular performance assessments including measuring of stress and burnout levels and a discussion on suitable coping strategies can also assist critical nurses to prevent the increase of burnout [50].

### Limitations of the study

Some limitations ought to be considered in generalizing the findings of the present study. The cross-sectional design and the use of self-reporting may increase statistical bias. One limitation of the present study was the collecting questionnaires after 2 days, due to the uncontrollability of all conditions, some nurses may refuse to provide real answers and hence, misreport.

Another limitation was the impossibility of controlling the predictive variables, such as nursing shortage, the lack of a work support and increased patient severity, perceived conflicts with patients or other staff and high workload, as mentioned in previous studies, it is recommended to conduct studies in this area. Also, the stressful life events and work-family conflict that may influence the results of burnout were not considered in this study.

## Conclusion

Among all dimensions of burnout, the lowest and highest mean belonged to the surgical and critical care unit, respectively. The critical care nurses have significantly higher levels of burnout compared to nurses in other wards.

In surgical wards, women showed the greatest level of burnout in all aspects whereas in medical wards, men showed the highest levels of burnout in all aspects indicating a significant difference between the two genders.

In surgical and medical wards, all aspects were significantly different in nurses holding a BS degree. There was a significant difference in all aspects of burnout according to marital status in surgical wards and married group had highest mean of burnout. Also, the difference was significant in “client-related burnout” in medical ward and being divorced exerted a significant effect on burnout level. Results showed there were a negative correlation between dimensions of “personal burnout” and “work aversion-related burnout” and “client-related burnout” with age and nursing experience in critical care wards.

In surgical wards, there were a positive correlation between dimension of “personal burnout” and nursing experience and also a positive correlation between dimension of “nature work-related burnout” with nursing experience and age. There are a negative correlation between dimension of “work aversion-related burnout” and age and nursing experience in medical wards.

## Data Availability

The datasets generated and analyzed during the current study are not publicly available due to an agreement with the participants on the confidentiality of the data but are available from the corresponding author on reasonable request.

## Abbreviations

EM: Emergency
HD: Hemodialysis
ICU: Intensive care unit
CBI: Copenhagen Burnout Inventory
P-CBI: Persian Copenhagen Burnout Inventory
ANOVA: Analysis of variance
SD: Standard deviation
BS: Bachelor of Science
MSc: Master of Science
